# Proposal and Validation of a New Index to Assess the Difficulty of Lower Third Molar Extraction

**DOI:** 10.3390/dj12050138

**Published:** 2024-05-09

**Authors:** Paula Hermida-Cabrera, Belén Lima-Sánchez, Vanessa Montoya-Salazar, Luis-Guillermo Oliveros-López, Pedro Alomar-Velasco, José-Luis Gutiérrez-Pérez, Daniel Torres-Lagares, María Baus-Domínguez

**Affiliations:** 1 Department of Stomatology, Faculty of Dentistry, University of Seville, 41009 Seville, Spain; paulahermidacabrera@gmail.com (P.H.-C.); belima97@gmail.com (B.L.-S.); vane843@hotmail.com (V.M.-S.); lgoliveros7@gmail.com (L.-G.O.-L.); jjlgp@us.es (J.-L.G.-P.); 2 Oral and Maxillofacial Unit, Quirónsalud Palmaplanas Hospital, 07010 Mallorca, Spain; doctoralomar35@hotmail.com; 3 Oral and Maxillofacial Unit, Virgen del Rocio Hospital, 41013 Seville, Spain

**Keywords:** difficulty indices, wisdom teeth, third molar extraction, validation, diagnostic procedure

## Abstract

There is no current consensus on the parameters that determine the difficulty of mandibular third molar extraction in terms of the time required, which is essential to prevent complications and optimize the time of the intervention. This study aims to obtain, using the mathematical method of multiple linear regression, an equation that allows estimating the extraction time of a lower third molar according to its complexity, as well as to validate this equation in a sample of external wisdom teeth. Methods: A prospective cohort study on a sample of patients of the Master of Oral Surgery of the University of Seville in which multiple linear regression coefficients were calculated with a subsequent validation study of the results in the sample of patients operated in the Hospital Palmaplanas of Mallorca. Results: The regression line obtained after applying the statistical methodology to the cohort of patients from the University of Seville obtained significant dependent variables such as depth, roots, and odontosection. Once applied to the cohort of patients from the Palmaplanas Hospital in Mallorca, a regression coefficient was obtained between the data received and the estimated 0.770. Conclusions: The formula proposed in this article presents significant validity in the prediction of the surgical time of extraction of the lower third molars included.

## 1. Introduction

The lower third molars show the highest proportion of eruption alterations, followed by canines, third molars, and maxillary second premolars [1]. 

About 27–68.6% of these eruptive problems are impactions [2], rising to 73% in the young adult European population [3]. This is why the surgical procedure of third molar removal is one of the most common in the daily practice of oral and maxillofacial surgeons, since the pathology derived from impacted third molars is extensive, affecting the patient in general and the oral cavity and adjacent teeth in particular [3,4]. 

Despite being one of the most frequent interventions, there is still controversy in the literature and a lack of consensus when evaluating the degree of surgical difficulty assigned to each clinical case [4]. Thus, many scales have been proposed over the years, as Bhansali et al. reported in a review published in 2021 [5].

Most of them evaluate the difficulty according to purely radiological and positional parameters, such as the well-known classifications of Winter et al. (1926) [6], Pell–Gregory (1933) [7], the WHARFE index (the first to relate surgical time to surgical difficulty) [8], the Pederson difficulty scale (1988) [9], and many others [5]. The latter, taken into consideration for years and cited in several relevant articles, has been modified by authors such as Kharma et al. [10], as it is specified in the review of Abdurrahman et al. [11], and Roy et al. [12] to add variables that accurately delimit the assessment of difficulty; these variables are the anatomy of the roots [11] and subjective clinical variables [12]. Even so, the specificity of 68.4% and sensitivity of 18.2% shown in the review of Abdurrahman et al. [11] of the Kharma scale can be explained by the fact that it still does not consider (as Pederson’s) relevant factors such as the flexibility of the patient’s cheek, bone density, or the degree of ankylosis possibly derived from the patient’s age [13]. 

This leads us to think that it is necessary to broaden the range of factors that are taken into account when speaking in terms of the difficulty of surgical intervention, such as third molar extraction, specifically mandibular, where it has been shown that even the level of anxiety of the patients before the intervention conditions infections and postoperative complications [14,15]. The preoperative stage is, therefore, essential to offer our patients the highest safety and quality in surgery [16]. 

It is at this surgical moment when the visual analog scale (VAS) comes into play, where the article by Sánchez Jorge et al. (2023) [17] shows how the importance attributed to each aspect varies depending on whether the operator is a student without surgical training, if the surgical training is at different stages of advancement, or if the operator is a qualified professional. As the individual becomes more professional, the importance resides more in the type of patient and their characteristics than in the intervention as such or the characteristics of the third molar in question [17], which is why this other aspect, such as the operator’s training, which in many cases has not been considered, should undoubtedly be considered. 

In these terms, several authors have tried to propose an index of difficulty of third-molar extraction. Juodzbalys et al. [18] calculated a classification based on anatomical and radiological findings, as well as a literature review. In this index, the main point was relating the third molar with its surroundings (apico-coronal position in relation to the alveolar crest and the mandibular canal; mesiodistal position in relation to the second molar and the mandibular ramus; and finally, buccolingual position in relation to mandibular lingual and buccal walls). This preoperative assessment provided a useful tool for planning the surgical operation. However, the proper author recognizes that this classification does not include several relevant parameters derived from the patient and the surgeon. Years later, Gay-Escoda et al. (2022) [19] proposed a new classification in their systematic review, considering not only radiological variables but also surgical variables (taking into account the point at which the surgeon’s training is) and those related to the patient (assessing physical and psychological factors), thus giving a broader approach to the idea of “scale of difficulty”.

Creating an index will not only provide professionals with the ability to be much more effective when planning surgery and optimizing time but also shed light on ways of minimizing postoperative sequelae. A recent clinical trial [20] shows how the difficulty of the intervention is related to the most common postoperative sequelae such as trismus, facial swelling, and pain, as well as its relationship with pharmacological treatment (prednisone), concluding that preoperative administration of prednisone is adequate to reduce postoperative sequelae after third molar surgery with conventional/moderate surgical difficulty.

Along the same lines, this study aims to find out which variables are most related to the complexity of lower wisdom tooth extraction, as well as to estimate the surgical time directly related to the difficulty of the wisdom tooth through a prospective cohort study on a sample of patients of the Master’s Degree in Oral Surgery of the University of Seville, in which multiple linear regression coefficients are calculated, with a subsequent validation study of the results in the sample of patients operated on at the Palmaplanas Hospital in Majorca.

## 2. Materials and Methods

### 2.1. Type of Study

Prospective cohort study approved by the Research Ethics Committee of the University of Seville with internal protocol code 1336-N-23 and which complies with all the guidelines of the Helsinki Declaration of Ethical Principles for Medical Research Involving Human Subjects.

All patients included in this study were informed of the study’s nature and gave informed consent for both participation in the study and the surgical procedure in question.

The only invasive procedure performed on patients was the extraction of third molars diagnosed with an absolute indication for extraction. 

### 2.2. Selection of Patients

The first cohort of patients, which was used to define the initial regression line, was composed of patients from the Faculty of Dentistry of the University of Seville who were diagnosed with third molar dysodontiasis and operated on in the Master’s Degree in Oral Surgery by different students of the master’s degree.

All patients met the following inclusion criteria: (1) patients with a diagnosis of third molar dysodontiasis; (2) patients with an absolute indication for third molar extraction; (3) patients with a detailed and complete clinical history; (4) patients with a radiological study (orthopantomography); and (5) patients with duly explained and signed informed consent.

The exclusion criteria for this study were as follows: (1) patients with severe or uncontrolled medical conditions such as cardiovascular disease, diabetes, blood clotting disorders, and any other condition that may have implications for oral surgery; (2) patients at high risk of nerve injury; and (3) patients who had experienced surgical complications during previous third molar extractions such as bone fractures and/or severe postoperative infections. These exclusion criteria were evaluated on an individual basis, taking into account the complete medical and dental history of the patient, as well as any specific considerations related to the lower third molar extraction procedure. After concluding the intervention, the following data were collected through a data sheet (Table 1).

### 2.3. Surgical Procedure

All lower wisdom tooth extractions were conducted using local anesthesia, specifically articaine 4% and epinephrine 1:100,000 (Ultracaín, Normon, Madrid, Spain). For semi-included or completely included wisdom teeth, a linear scalloped or bayonet mucoperiosteal flap was designed to provide optimal access to the surgical site. Ostectomy was performed using a number 8 tungsten carbide round bur and handpiece with copious irrigation. A Lindemann drill and turbine were utilized for odontosection if needed. 

Luxation and extraction of the wisdom teeth were accomplished using different widths of straight extractors and Winter extractors as necessary. 

After extraction, the alveolus was thoroughly irrigated to ensure the complete removal of any bone or tooth fragments, with a particular focus on the follicular sac. The distal face of the second molars in contact with the surgical site was sutured with Gracey Curettes 13/14 to guarantee proper periodontal insertion in the area. 

Sutures were performed using Mayo needle holders and Supramid Aragó 4/0 or 5/0 TB12-CT 16 mm 3/8 nonabsorbable sutures. 

All patients received written and verbal postoperative instructions and were scheduled for a follow-up visit and suture removal after one week. The prescribed pharmacological regimen consisted of 400–600 mg ibuprofen every 8 h for 5–7 days, in combination with 650–1000 mg paracetamol every 8 h for 5–7 days. For surgeries lasting more than 2 h, major ostectomy procedures, or for patients with active infections or altered immune systems, 500 mg of amoxicillin was prescribed every 8 h for 3 days, with a review on the third day to determine whether or not to continue with the antibiotic regimen, in accordance with the recommendations of the National Antibiotic Resistance Plan and the Antimicrobial Therapeutic Guide of the Spanish National Health System.

### 2.4. Statistical Analysis

The data on lower jaw surgery were received on the cards designed to collect the different extraction characteristics. All the data were coded in a table with SPSS 9.0 (IBM, Armonk, NY, USA), on which statistical methods were applied using the software. 

A complete descriptive study was carried out detailing all the variables.

Multiple linear regression using the backward method was used to estimate the time in minutes required for the intervention from the values of various continuous or discrete variables.

It is indicated with the usual format (*p* < 0.05; *p* < 0.01; *p* < 0.001, *p* < 0.0001, and *p* < 0.00001); the lower the figure, the higher the significance.

### 2.5. Validation

For the validation study, the participants were patients diagnosed with third molar dysodontitis at the Hospital QuirónSalud Palmaplanas in Palma de Mallorca, Spain and operated on by a single expert surgeon. 

For the crossover between qualitative variables, the Chi test^2^ was used. To determine the groups that make the difference, we used Haberman’s corrected typified residuals, which allowed us to obtain the significance of the cells independently. This significance implies that the percentage of the cell is statistically different from that corresponding to the total of the sample.

The Mann–Whitney U test was used for the crossover with respect to the numerical variables since they do not follow a normal distribution.

Spearman’s correlation coefficient was used for correlations.

## 3. Results

### 3.1. Participants and Characteristics

The present study’s sample consisted of 30 patients from the University of Seville, on whom coefficients for multiple linear regression were calculated, and 71 patients from the Palmaplanas Hospital in Mallorca, where the results obtained from the University of Seville sample were validated (Table 2).

### 3.2. Variables Related to the Lower Wisdom Teeth Operated on

The data of the variables related to the lower wisdom teeth operated on in the sample are shown in the table below (Table 3). 

### 3.3. Regression Equation

Y is the total time of the intervention. C is the constant of the model (column B for the Constant row (Table 4)). B_i_ is the value in column B for each variable in the model, and X_i_ is the value of the independent variable to which B corresponds.

Y = C + B_1_ ∗ X_1_ + B_2_ ∗ X_2_ + B_3_ ∗ X_3_ + …

Table 4 summarizes the coefficients for each variable obtained from the application of the regression equation once the surgeries were performed on the sample of patients from the University of Seville. 

**Figure 1:**
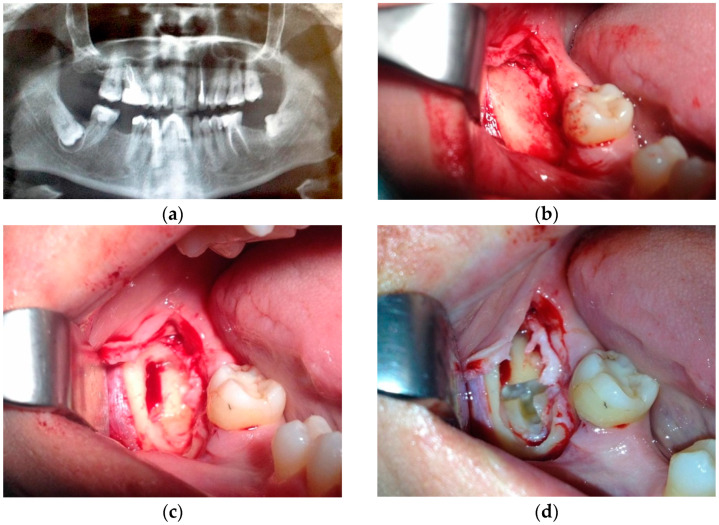

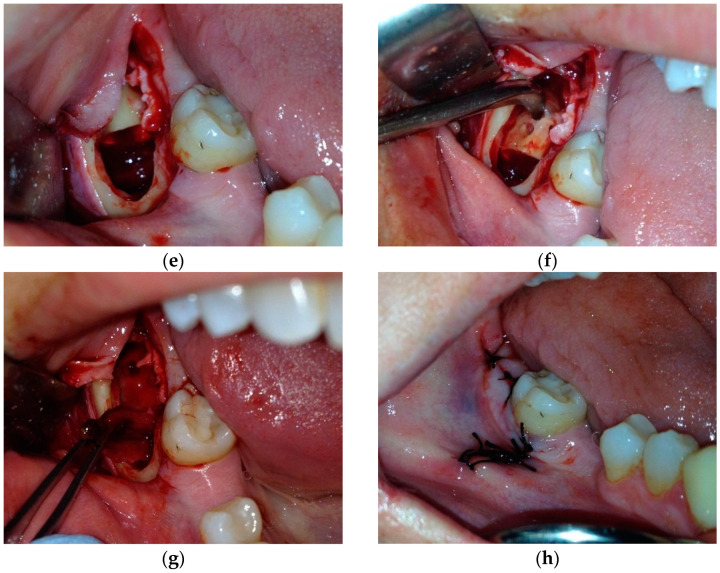
Extraction of a lower level C deep wisdom tooth with more than 2/3 of fused root and in need of odontosection; Y = C + B1 ∗ X1 + B2 ∗ X2 + B3 ∗ X3 = 22.971 + 0.237 ∗ 3 + 0.376 ∗ 1 − 0.524 ∗ 2 = 23 min of extraction: (**a**) orthopantomography; (**b**) full thickness flap; (**c**) start of ostectomy; (**d**) crown odontosection; (**e**) visualization of roots inside the socket; (**f**) root luxation; (**g**) root extraction; (**h**) suturing.

X_i_, which corresponds to the value of the independent variable for each B_i_, presents a value from 1 to 3 according to its difficulty index (based on the indexes already published), with 1 being the most straightforward situation and 2 or 3 (depending on which variable) the most complex for the extraction of a lower third molar (Table 5) (Figure 1).

The following table shows the prognosis’s statistical values and the difference in absolute terms between the actual time taken to extract the third molar and the estimated time calculated through the regression equation (Table 6).

### 3.4. Validation

To validate the equation, an estimate was made on the data of patients operated on at the Palmaplanas Hospital in Mallorca (71 patients) using the regression equation defined from the data of the University of Seville (Table 4) with a correction to establish a minimum performance of two minutes, since the mathematical equation estimates negative values for the interventions considered to be the simplest (Table 7).

To conclude, a table summarizing the model is attached (Table 8).

As can be seen, after applying the coefficients obtained from the data of the patients operated on in Seville to those in Mallorca, an r of 0.770 is received, indicating a positive correlation very close to 1 (highest value for r).

To clarify and facilitate the interpretation of the statistics it can be explained that the regression equation was applied, and a series of coefficients were obtained for each variable (Table 4). The constants (B) are adjusted by a computer program that searches for the best coefficient for all variables. In this way, only the variables that contributed statistical significance with a value of *p* < 0.001 were included. These variables are as shown in Table 4 and Table 5: depth of the wisdom tooth (classified as A, B, C of Pell–Gregory), roots (fused and/or multiple), and whether or not odontosection was performed (specifications of the variables in Table 3). 

Avoiding that this study be based solely on internal validity (30 patients from Seville), the validation of the results was carried out with a sample of 71 patients from Mallorca, applying the multiple linear regression equation and obtaining a correlation coefficient (r) equal to 0.770, with the highest value being 1.

## 4. Discussion

Currently, there is no consensus index useful for evaluating the surgical difficulty involved in third molar extraction and estimating the total time that the intervention may entail. This lack of consensus can be explained by the numerous variables that condition the intervention concerning the wisdom tooth, the patient, and the operator, and the operator’s subjectivity in the case is worth considering. 

The operator’s ability to predict the intervention’s difficulty is likely by clinical experience. Sanchez-Jorge et al. [17] conclude this in their article published in 2023, where, using the visual analog scale (VAS), they determine that those individuals with no previous surgical training perceive more incredible difficulty in the intervention than those professionals with postgraduate training. At the same time, the types of factors taken into account by the two groups vary, with those related to the patient being more relevant for the professionals, in contrast to the surgical factors to which dentists without postgraduate training attach more importance. Along the same lines, studies such as that of Pippi et al. [21] seek to compare the difference in perception of the difficulty of the intervention before and after performing it between groups with different levels of training, finding as a result that those with specialized surgical training did not change their opinion after performing the intervention, while Barreiro Torres et al. [22] state that there is no correlation with the preoperative and postoperative idea, since there is a tendency to underestimate the intervention, mainly among the more highly trained groups, this correlation being 38.7% for maxillofacial surgeons, 45.1% in oral surgeons, and 31.9% for general dentists. He thus concludes that regardless of clinical experience, the visual analog scale and the use of panoramic radiographs do not help predict the difficulty of the intervention, irrespective of the degree of training [19,22]. Ferrus-Torres et al. [23], on the other hand, determined in their study that the predictive capacity of novice surgeons tends to be more erroneous, so the degree of training does influence predicting the level of difficulty, in agreement with the findings of Sánchez-Jorge et al. [17]. In the present study, all the interventions were performed by Master’s degree students with the same level of training in Seville. In Mallorca, an expert surgeon performed the surgeries.

On the other hand, the anatomical factors and those related to the wisdom tooth in question and the patient are of utmost relevance to be evaluated. In the 1920s and 1930s of the last century, two classifications were published that are still in force today: Winter’s (1926) [6] and Pell–Gregory’s (1933) [7]. The first one classified the wisdom teeth into five positions according to their angulation; the second one classified them according to the position of the wisdom tooth concerning the ascending mandibular branch and the occlusal plane [6,7]. 

Over the years and to complete the information on the intervention, MacGregor published, in 1976, the first index that relates the difficulty of the intervention with the time spent on it through the use of orthopantomography and the WHARFE index, which we cover later [8]. On the other hand, and arguably just as widespread, we find Pederson’s classification [9], variations of which have been presented by authors such as Kharma et al. [10] and Roy et al. [12]. 

Roy et al., in their study published in 2015, underline the time spent as the determining factor in discussing the difficulty of wisdom tooth surgery. The Kappa concordance is 89% in the new proposal, which is welcomed in the article [12], compared with the 66.5% Kappa of the Pederson index. The modifications are based on aspects of the patient that Roy et al. consider key to speaking in terms of difficulty, such as buccal opening, tongue size, mandibular external oblique ridge anatomy, cheek flexibility, root width, and root morphology. Therefore, this new proposed index is considered a better predictor of difficulty. In our study, these kinds of aspects of patients were not included from the beginning, so it cannot be determined if they may have shown enough clinical and statistical significance to be included in the formula. 

Regarding Kharma’s proposal [10], an article by Al-Samman et al. [11] evaluated its predictability, showing a sensitivity of 18.2% and a specificity of 68.4%. Still, there was no significant association between the scores obtained on the scale and the duration of the intervention, so the study concludes that this scale is unreliable for determining the difficulty of this type of intervention (understanding that the difficulty is governed by the time spent on it).

Despite these classifications being widely known and used, it cannot be denied that they are lacking in providing relevant information, and considering they are 2D-based classifications, many aspects of the patient are ignored. In these terms, Selene Barone et al. [24] published a three-dimensional study to correlate the impaction of the lower third molar and gonial angle by performing a morphometric analysis of the mandible through CBCT. The third molar was analyzed in the sagittal, coronal, and axial planes. A reduced gonial angle value was significantly associated with a deeply affected molar in the ramus, and at the same time, a progressive decrease in the gonial angle meant a more horizontal position of the molar closer to the mandibular canal (*p* < 0.05), and finally, a lower gonial angle showed a reduced retromolar space with a more complex impacted third molar (*p* < 0.05), so there is a correlation. In contrast, an article published by Jeevitha et al. [25] finds no existing correlation between both aspects, although it is relevant to highlight it used 2D radiographs. Multiple recent studies compared other aspects of craniofacial morphology, concluding in a similar way: facial types influence the kind of impaction, for example, the growth potential of the mandible in brachyfacial types may provide sufficient space for third molar eruption, which is a more common impaction of the molars in dolichocephalic [26], and individuals with increased maximal cranial width and decreased anterior facial height have a higher risk of impaction of third molars in the mandible [27].

As it has been developed above, the operating time is, therefore, a recurring theme in all the studies in this field, and it is the central topic that concerns our study. An article by Alvira-González et al. [13] relates the operating time and VAS with different classifications and aspects of the patient to assess the difficulty of the intervention. Patient weight, ankylosed roots, the need for osteotomy and odontosection, and the Pell and Gregory IIIC classification, or in Winter’s classification, distalized and vertical, increase operation time with a *p* < 0.05. Facial pattern, on the other hand, does not seem to influence the intervention time, as does height or gender. In their article, Tenglikar et al. [15] conclude that factors such as buccal opening, external oblique ridge, and root morphology are determining factors that vary the operation time [13,16,28], variables that have not been considered in the present study. Root morphology is a factor that includes the WHARFE index, which is analyzed and compared with Pederson’s in a study published in 2020 [29,30], where it is concluded that the WHARFE index is more reliable and accurate than Pederson’s, which is supported by other previous publications [30,31]. 

Bhuju et al. [15] analyzed different patient variables and their relationship with the duration of treatment using Pearson’s χ^2^ test, concluding that one of the factors that most affect the prediction of the duration of the intervention is gender, with a *p* = 0.043, and that age could have an influence due to the difference in densities and more developed bone disorders in older patients but did not obtain statistically significant results. Previous studies, however, did show age as an aspect that increases intraoperative and postoperative complications; one published in 2007 [32] used a multiple regression model to conclude that patients >25 years old were associated with a higher rate of complications. On the other hand, Bhuju et al., in their study, reflected on the impact of a patient’s fear and anxiety on intervention, something that is also reflected in the publications of Le et al. in 2021 [14] or Barbosa Bisson et al. in 2022 [33], where they highlight the importance of these factors together with stress, not only perioperatively and intraoperatively but also at the time of scarring. Regarding the postoperative period of wisdom teeth, an article was published at the end of 2023 by Sainz de Baranda et al. [34], where the Pederson scale is considered helpful in predicting the postoperative period of patients (*p* < 0.001), the greater the degree of difficulty, the higher the levels of C-reactive protein, fibrinogen, and interleukin 6 (IL-6), as well as the greater the number of postoperative clinical parameters (pain, inflammation, and trismus) [34,35].

Linked to the above, the importance of developing consensual difficulty indexes and scales, as has been attempted for years [6,7,8,9,11,36,37] and as reflected by Gay Escoda et al. [19], is precisely to control and prevent the intraoperative and postoperative phase of this type of intervention, preparing us for possible difficulties and complications.

As has been developed and highlighted throughout the discussion, time is considered the reference to determine the difficulty of the intervention. Predicting the surgical time before surgery allows for optimizing time and helping professionals face each case individually and referring when necessary.

In these terms, the present study proposes a method that has not been described before in the literature. After developing the statistical analysis, it is determined that between the variables presented and statistical significance, there is a positive correlation, and as the value of one variable increases so does the other; this relation and capacity to adjust an intraoperative time of the intervention has not been raised in recently proposed indexes, such as those discussed in the discussion, and even in those in which the time factor is mentioned, there is no mathematical prediction [11,12,15,18]. 

It is important to note that there are some limitations that need to be taken into account when interpreting the results of this study related to the extraction of impacted wisdom teeth, despite the efforts made to understand various aspects. 

One of the main limitations is that although multiple relevant variables have been evaluated and considered, such as depth, spatial position, position relative to the second molar, need for ostectomy, odontosection, and root morphology, the possibility of complications during the extraction of included wisdom teeth has not been explicitly included in the multiple linear regression model. 

While these variables provide an understanding of the factors that may influence extraction time, it is important to note that intraoperative complications such as excessive bleeding or other unexpected complications can significantly affect the duration of the procedure, which has not been addressed in this study. 

Future research should consider the inclusion of additional measures to assess and mitigate complications during the extraction of included wisdom teeth, which could improve the precision and clinical utility of predictive extraction time models.

Another limitation of this study is the difference in the experience of the operators during the extraction of included wisdom teeth. A group of wisdom teeth was operated on by students of the Master of Oral Surgery at the University of Seville under teaching supervision, while another group was operated on by an experienced oral surgeon. This difference could have influenced several aspects of the procedure, including technical skill, clinical decision making, and the management of possible complications, which could have affected the results obtained.

Additionally, the present study does not consider certain patient-derived aspects, such as how facial morphology can affect the difficulty of this type of procedure or even how ethnic characteristics can influence surgical intervention.

For these reasons, a validation study of the regression line obtained in different universities and participants from other regions may be interesting to test the clinical significance of the proposed formula.

## 5. Conclusions

Taking into account the limitations of the study, the formula presented and the variables included in it for lower wisdom teeth have clinical and statistical relevance when predicting the surgical time before wisdom tooth surgery. Thus, they help the operator optimize surgery and assess the possibility of referring certain cases.

However, more studies of this type should be conducted to test the validation of the equation in different samples. 

## Figures and Tables

**Table 1:** Intervention data sheet.

INTERVENTION TIME	________ MINUTES
**IDENTIFICATION**	Left Right
**SPATIAL RELATIONSHIP**	Mesioangular Horizontal/transverse Vertical Distoangular
**DEPTH**	Level A Level B Level C
**BRANCH/2M DISTAL FACE RATIO**	Class I ClassII Class III
**BONE AND MUCOSAL INTEGRITY**	Partially covered by mucosa Partially covered by bone and mucosa Totally covered only by mucosa, not by bone Covered by mucosa and partially by bone Completely covered by mucosa and bone
**ROOTS**	More than 2/3 merged More than 2/3 separated or less than 1/3 separated More than 2/3, multiple
**FOLLICLE SIZE**	>1 mm 0 mm
**ACTIONS (more than one box can be checked)**	Osteotomy Odontosection Sutura Simple exodontia

**Table 2:** General variables by center.

Variables	Categories	Seville		Mallorca		Sign.
		Frequency	Percentage	Frequency	Percentage	
Sex	Man Woman	17	56.7	27	38.0	quasi
		13	43.3	44	62.0	
Age (categorized)	Up to 22 years old	12	42.9	22	29.3	
	From 23 to 29 years old	8	28.6	19	25.3	
	From 30 to 39 years old	5	17.9	13	17.3	
	40 or more years	3	10.7	21	28.0	
Ostectomy	Yes	25	80.6	42	56.0	<0.05
	No	6	19.4	33	44.0	
Odontosection	Yes	24	77.4	31	41.3	<0.001
	No	7	22.6	44	58.7	
Suture	Yes	28	90.3	66	88.0	
	No	3	9.7	9	12.0	
Simple exodontia	Yes	3	9.7	32	42.7	<0.01
	No	28	90.3	43	57.3	

**Table 3:** Variables related to the lower third molars operated on in the sample.

Variables	Categories	Seville		Mallorca		Sign.
		Frequency	Percentage	Frequency	Percentage	
Spatial relationship	Mesioangular Horizontal/Angled	8	25.8	30	40.0	
		9	29.0	9	12.0	
	Vertical	13	41.9	32	42.7	
	Distoangular	1	3.2	4	5.3	
Depth	Level A	14	45.2	41	54.7	
	Level B	12	38.7	26	34.7	
	Level C	5	16.1	8	10.7	
Ratio branch/distal face 2M	Class I	14	45.2	41	54.7	
	Class II	14	45.2	26	34.7	
	Class III	3	9.7	8	10.7	
Bone and mucosal integrity	CParc. Mucosa	16	51.6	34	45.3	<0.05
	CParc. Hue. Y Muc.	7	22.6	3	4.0	
	CTot. Muc. No Hue.	1	3.2	4	5.3	
	C Muc and Parc. Hue	4	12.9	18	24.0	
	CTot. Muc. and Bone	3	9.7	16	21.3	
Roots	>2/3, merged	14	45.2	41	54.7	
	>2/3, sep or <1/3	14	45.2	32	42.7	
	>2/3, multiple	3	9.7	2	2.7	
Follicle size	>1 mm	22	71.0	49	65.3	
	0 mm	9	29.0	26	34.7	
Difficulty index (categorized)	Not very difficult	13	41.9	33	44.0	
	Difficult	16	51.6	34	45.3	
	Very difficult	2	6.5	8	10.7	
Difficulty index (dichotomous)	Not very difficult	13	41.9	33	44.0	
	Difficult or Very Difficult	18	58.1	42	56.0	

**Table 4:** Coefficients for each variable from the cases operated at the University of Seville.

Variable	Non-Standardized Coefficients		Coef. Est.	t	Sig (*p*)
	B	Error	Beta		
(Constant)	22.971	6.410		3.584	0.001
IQ: Depth	3.810	2.155	0.237	1.768	0.088
CI: Roots	6.751	2.403	0.376	2.810	0.009
Odontosection	−14.641	3.448	−0.524	−4.247	0.000

**Table 5:** Value of X_i_ for each variable as a function of extraction difficulty.

Variable	Categories	Value of X_i_
Depth	Level A	1
	Level B	2
	Level C	3
Roots	>2/3, merged	1
	>2/3, sep or <1/3	2
	>2/3, multiple	3
Odontosection	No	1
	Yes	2

**Table 6:** Statistical values of the forecast and the difference in absolute terms between actual time and estimation.

Forecast		Forecast Error (Actual Value—Estimate)				
Media	Standard Deviation	Media	Standard Deviation	Q1	Q2	Q3
22.65	9.14	0.00	−2.73	5.89	2.70	−0.55

The mean is 0 due to the construction of the model itself (the regression line is constructed so that the mean of the errors is minimal).

**Table 7:** Statistical values of the forecast and the difference in absolute terms between the actual time and the estimate were applied to the Mallorca data.

Forecast		Forecast Error (Actual Value—Estimate)				
Media	Standard Deviation	Media	Standard Deviation	Q1	Q2	Q3
15.67	10.88	−0.94	6.86	−5.00	0.19	2.75

For the estimation of the Mallorca data, the regression equation defined from the Seville data (Table 3) was used with a correction, establishing a minimum performance of two minutes (the mathematical equation estimates negative values for the interventions considered the simplest).

**Table 8:** Summary of the model.

R	R Square	Adjusted R-Squared	Standard Error of the Estimate
0.770	0.593	0.548	7.981

## Data Availability

The raw data supporting the conclusions of this article will be made available by the authors upon request.
